# Fears from the past? The innate ability of dogs to detect predator scents

**DOI:** 10.1007/s10071-020-01379-y

**Published:** 2020-04-08

**Authors:** Lydia Samuel, Charlotte Arnesen, Andreas Zedrosser, Frank Rosell

**Affiliations:** 1 Department of Natural Resources, University of Derby, Kedleston Road, Derby, DE22 1GB Derbyshire UK; 2 Department of Natural Sciences and Environmental Health, University of South-Eastern Norway, Bø, Telemark Norway; 3 Department for Integrative Biology, Institute for Wildlife Biology and Game Management, University for Natural Resources and Life Sciences, Gregor Mendel Str. 33, 1180 Vienna, Austria

**Keywords:** *Canis familiaris*, Detection-dogs, Scent, *Ursus arctos*, *Lynx lynx*, Innate behaviour

## Abstract

Throughout the animal kingdom, antipredator mechanisms are an evolutionary driving force to enable the survival of species classified as prey. Information regarding a predator’s location can be determined through chemosensory cues from urine, faeces, visual and/or acoustic signals and anal gland secretions; and in several lab and field-based studies it has been seen that these cues mediate behavioural changes within prey species. These behaviours are often linked to fear and avoidance, which will in turn increase the prey’s survival rate. In many studies dogs (*Canis lupus familiaris*) have been used as a predator species, however, no research has addressed a dog’s innate ability to detect predator scents, hence the rationale behind this study. We assessed the innate ability of the untrained domestic dog to detect faecal scents of wild Eurasian brown bear (*Ursus arctos arctos*) and European lynx (*Lynx lynx*). The study monitored 82 domestic dogs across the UK and Norway. The dogs were exposed to the two predator faecal scents from Eurasian brown bear and European lynx, a herbivore faecal scent of Eurasian beaver (*Castor fiber*) and water control. Measurements were taken upon the time spent within a 40 cm radius of each scent and changes in the dog’s heart rate when within this 40 cm radius. We found dogs spent a decreased length of time around the predator scents and had an increased heart rate in relation to their basal heart rate. We conclude that dogs can innately sense predator scents of brown bear and lynx and elicit fear towards these odours, as shown through behavioural and physiological changes.

**Electronic supplementary material:**

The online version of this article (10.1007/s10071-020-01379-y) contains supplementary material, which is available to authorized users.

## Introduction

Antipredator mechanisms act as an evolutionary driving force within the animal kingdom (Brodie et al. 1991). The predator–prey arms race has driven adaptations in defence and avoidance, and these behavioural changes in turn increase prey survival rate (Barrio et al. 2010). Information regarding a predator’s location can be determined via chemosensory cues (Kats and Dill 1998), revealing the presence of predators or their recent home ranges (Head et al. 2002). It is of great importance that prey can easily identify these cues (Mitchell et al. 2015) to enable their survival. Typical odours are derived from predator’s urine, faeces, fur and anal gland secretions (Apfelbach et al. 2005). These chemical cues have been documented to mediate interspecific reactions enabling prey to detect adverse cues from predators they have never previously been in contact with (Ferrari et al. 2010).

Potential predator detection in many cases may be experience-based (Salo et al. 2007). A study upon the Tammar wallaby (*Macropus eugenii)* and the Red-necked pademelon *(Thylogale thetis)* revealed predator naïve individuals did not respond to predator odours, whereas the individuals which were wild and had predator experience showed very different behavioural responses (Blumstein et al. 2002). Studies upon predator naïve moose (*Alces alces*) alternatively suggest that these moose react to the scents of unknown predators (Berger et al. 2001). Prey may identify predators based upon metabolites of sulphurous molecules derived from meat within the carnivore’s faeces, which in turn triggers an innate reaction (Nolte et al. 1994). For example, reef fish have been observed to exhibit a lower level of settlement on reefs containing individuals with a highly piscivorous diet (Dixson et al. 2012). Studies upon the North American beaver (*Castor canadensis)* have shown a decreased feeding behaviour to kairomones (a chemical omitted by an organism, which mediates interspecific interactions) of both native predators and African lions (*Panthera leo)* faeces (Engelhart and Muller-Schwarze 1995). Similarly, research upon foraging activities of *Castor fiber* suggested that the presence of predator odours such as the red fox (*Vulpes vulpes*), Eurasian lynx (*Lynx lynx*) and brown bear (*Ursus arctos*), resulted in a decreased foraging activity (Rosell and Czech 2000). Research further suggests predator recognition may also be dependent upon phylogenetic relationships (Sih et al. 2010); prey which is more closely related to predators are recognised more efficiently as threatening by the prey species (Mitchell et al. 2015).

Olfaction is an important sense in domestic dogs (*Canis lupus familiaris*) to enable precise chemosensation (Olender et al. 2004), enabling them to generalise scents. Dogs have 1094 olfactory receptor genes (Adams and Johnson 1994); making them extremely efficient at detecting the presence of odours, being able to smell compounds at concentrations as low as one part per trillion (Dias and Ressler 2014). Studies evaluating scent recognition by dogs towards predators have often utilised trained detection dogs (Smith et al. 2003; Wasser et al. 2009). However, to our knowledge, no studies have yet investigated the innate ability of dogs to recognise scents of potential predators. It is well documented that in stressful and fear-induced situations or activities several physiological systems are activated in dogs. Both behavioural and heart rate changes are thus useful indicators to assess emotional stress reactions, including those of dogs (Palestrini et al. 2005; Beerda et al. 1998), due to interactions in the central nervous system and neuroendocrine system (Hydbring-Sandberg et al. 2004).

We used an experimental design where we presented dogs with a predator scent [i.e., faeces from the brown bear (*Ursus arctos*) or Eurasian lynx (*Lynx lynx*), a herbivore scent (faeces from Eurasian beaver (*Castor fiber*)], and a control scent (water). We measured the reaction of dogs to these scents as the length of time spent within a 40 cm diameter of the scent. In addition, we also measured the heart rate [in beats per minute (bpm)], in relation to the different scents during the same trial. We hypothesise that the scents of the predators will lead to overall stronger reactions in the dogs compared to the herbivore and control scents source. We predict that dogs will spend less time within a 40 cm diameter of the scent source compared to the herbivore and control scents. Furthermore, we predict that dogs will have a higher heart rate when within a 40 cm diameter of the predator scents source compared to the herbivore and control scents source. To evaluate if a dog’s individual characteristics affected the above measurements, we controlled in the analyses for the effects of age, sex, weight and if a dog was trained as a hunting dog.

## Methods

Bear scat samples (*N* = 55) were collected during an individual-based long-term project studying wild bears in Scandinavia (Stenset et al. 2016, Zedrosser et al*.* 201; Swenson et al*.* 1994) and beaver samples (*N* = 55) were collected during an individual-based long-term project studying wild beavers in Norway. Brown bear scats had been collected out in the field during fall 2015/2016 with a diet consisting mainly of berries and vegetation (Zedrosser, unpublished data). Age of scats varied between a couple of days old and up to less than 2 weeks old. Beaver scats were collected during fall 2015/2016, having a diet of aquatic vegetation and tree bark consisting mostly of willow (*Salix caprea*) and birch (*Betula pubescens*) (Rosell 2018). The beaver scats were collected from the captured animal. Scats (*N* = 27) from captive Eurasian lynx were provided from; Newforest Wildlife Park—Ashurst, The Big Cat Sanctuary—Ashford, ARK Wildlife Park—Boston, Drayton Manor Zoo—Mile Oak, Dartmoor Zoo—Sparkwell, Five Sisters Zoo—Wet Calder, Dudley Zoo—Dudley, Shepreth Wildlife Park—Shepreth, Birmingham Wildlife Conservation Park—Birmingham and The Royal Highlands Wildlife Park—Kincraig and all collected from the ground. The lynx were fed a mixed diet of small mammals, chicken, game birds, cattle, horse, venison and fish. All scats were frozen immediately after collection and stored at − 20 °C until use. All scats used in the study were collected from the ground from live animals. All methods were performed in accordance with the relevant guidelines and regulations of the University of South-East Norway. Further approvals from other ethics committees or ethics boards were not needed. No animals experienced anaesthesia, euthanasia or any kind of sacrifice as a part of this study. Non-invasive sampling was used for sample collection thus no permits were required. Further, no permits were required to enter the areas where these samples were collected.

## Experimental design

**Fig. 1:**
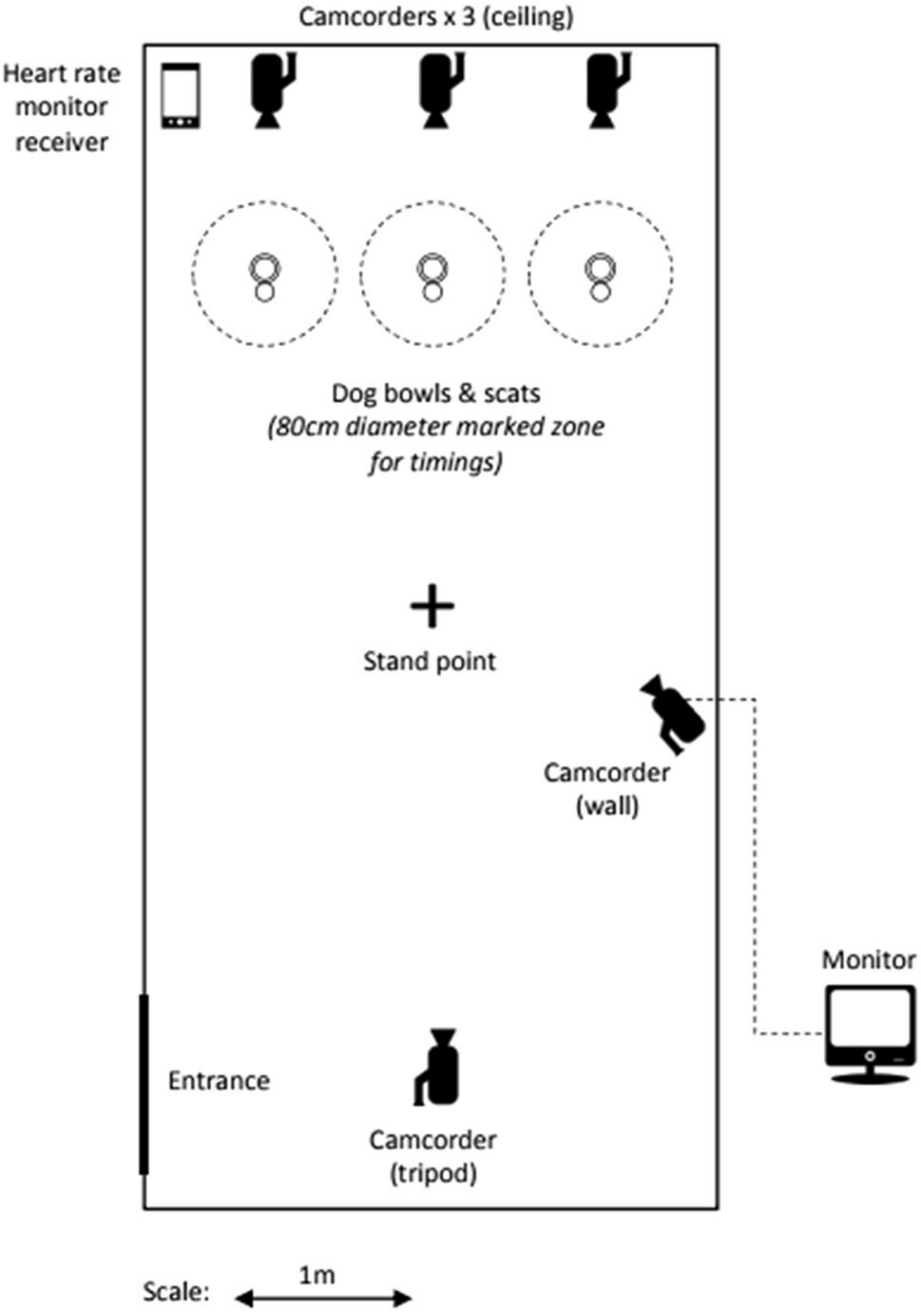
Layout of the trial set-up used to measure the dogs' responses to the tested scents: predator (bear of lynx) scat, herbivore (beaver) scat and control (water)

We simultaneously presented individual domestic dogs (*N* = 82) with a predator scat (faecal scent) (either brown bear or lynx), a herbivore scat (faecal scent) (Eurasian beaver) and a control scent (water). Trials using bear faecal scents (*N* = 55) were conducted from 12 to 28th June 2017 and 15 to 17th August 2017 in an unused barn (3.2 m × 6.5 m, Fig. 1) in Bø in Telemark, Norway. Trials with lynx faecal scent (*N* = 27) were conducted from 1 to 27th July 2017 in an unused outbuilding (2.56 m^2^ × 5 m^2^) in Ashby-de-la-Zouch, Leicestershire, England.

Pre-study information was determined upon each dog. The information included the dogs; weight (kg), breed, sex, age and hunting experience (see online resource). A trained hunting dog was defined for the purpose of the study, as a dog which has been or is currently used to hunt alongside humans.

Owners were instructed not to feed their dogs the morning of testing. Dogs were presented with three visually identical bowls in each trial. Each bowl contained a mixture of dry dog food and sausage meat. The amount of dog food presented was half the recommended daily weight of dry dog food in relation to the body size of the dog, spread evenly across the three bowls. The sausage meat was added as an additional incentive, in amounts adjusted to the weight of the dog. Placed in front of each bowl was a Petri dish (100 × 15 mm, FB0875713, Fisherbrand™) with the different scents concealed by a paper circle containing 40 holes to ensure diffusion of the scent yet concealment from the dog’s sight. The scat and Petri dishes were prepared away from the experimental room ten minutes before the start of a trial. A scat (ca 10 g) was placed onto the Petri dish using latex gloves and the paper sealed over the Petri dish using tape. The scat for each trial was randomly selected by an individual independent from the study to ensure that the study was performed double-blind (Kardish et al. 2015). The positioning of the scat containing Petri dish in front of each bowl was determined by the use of a random number generator and positioned by an individual independent from the study. Each scat was only used once to avoid pseudoreplication (Kroodsma et al. 2001) and each scat was non-mixed, each being from an individual animal.

The bowls were placed 90 cm apart and 1 m from the back wall, opposite to where the dogs entered, with a radius of 40 cm clearly marked around each bowl. Three camcorders were hung from the ceiling above each of the three bowls alongside a heart rate receiver (smartphone) (Fig. 1). The experimenter was positioned outside of the room when a trial was carried out, watching each trial from a monitor connected to a camcorder positioned at the back of the room.

Each dog was fitted with a Polar H7 Bluetooth heart rate monitor (Essner et al. 2012; Craig et al. 2017) and was allowed to enter into the experimental room just once. The monitor was moistened by electrode gel to ensure conductivity and placed around the dog’s sternum. The heart rate was monitored and recorded via a smartphone throughout the trials. A time of five minutes was designated to determining each dog’s basal heart rate which was taken as a mean. This mean was determined for each dog outside of the study room in the presence of the owner and experimenter prior to each trial when they were perceived to be most relaxed.

Participants were scheduled to arrive in sequence, so no dogs nor owners witnessed trials previous to their own. No other dogs or individuals were present in the room while a trial was run. The owner stayed with the dog to lessen anxiety and if necessary was allowed to give the command to roam and eat at the start of the trial, but was asked to remain passive and avoid eye contact with the dog during the course of the trial. The dog was kept on an extendable lead to allow free roam. The owner walked with their dog to the standpoint (centre of the study room) and remained there, so the dog alone decided the order of bowls it visited. Recordings were taken including the time when the dog directed attention towards the sample through being within the 40 cm radius of the bowl (which was clearly indicated around each bowl). The time was started as soon as the dog stepped into the 40 cm radius of a bowl and ended when they left, this occurred for each of the different bowls. Heart rate data were collected in the same manner, the heart rate data was taken from when the dog was in the 40 cm radius of each bowl. The end of a trial was determined when the dog had visited all three scent bowls at least once and had stopped directing its attention to the bowls.

In between each trial, the experimental room was sprayed down with 7% vinegar spray to remove scents left by previous dogs (Arendash et al. 2001, 2006) and left to dry. Due to vinegar being an aqueous solution of acetic acid it easily bonds with volatile molecules and thus was used to remove the scents of the previous dogs. The study rooms were further ventilated to aid in diffusion, with doors additionally being left open and both study areas containing concrete flooring.

### Statistical analysis

We used linear mixed models (LMM) to analyse if time spent and mean heart rate within the 40 cm radius of the scents were affected differently by the scent of a predator (bear or lynx), herbivore (as factor beaver), or a control (water). The scent types were included as factor variables in the analysis (bear/lynx = 0, beaver = 1, control = 2). In addition, we controlled for a dog’s sex (factor, with levels female = 0, male = 1), weight, age, if it was used as a hunting dog (factor, with levels, no = 0, yes = 1), and if the dog had previous experience with bear scent (factor, with levels, no = 0, yes = 1). None of the dogs participating in the lynx trials had previous experience with lynx or lynx scent, therefore we did not control for this in our analysis. We used the trial number as a random variable. Trials with bears scent were analysed separately from trials containing lynx scent, because these trials were carried out in a different experimental setting and environment (i.e., barn in Bø, Norway, outbuilding in Leicestershire, United Kingdom). We carried out a backwards selection procedure until the final model consisted only of significant or suggestive terms. We used the package *lme4* (Bates et al. 2015) in the statistical software R 3.4.2 (R Core Team 2017).

## Results

A sample of 55 dogs was used for the bear trials, with a mean age of 5.6 ± 3.7 (SD) years (range 4 months–15 years). Of these dogs, 49 were pure breeds and six mixed breeds; 19 dogs had hunting experience, and 18 dogs were classified as having previous experience with bear scents through direct encounters, hunting or used in a pilot study with bear scents. For the lynx trials, a sample of 27 dogs was used, with a mean age of 5.2 ± 3.6 years (3 months–11 years). Of these dogs, 19 dogs were pure breeds and eight were mixed breeds. Six of the dogs had hunting experience but none had previous experience with lynx or their scent. Due to the low sample sizes, we did not include breed into further analyses.

### Time

**Fig. 2:**
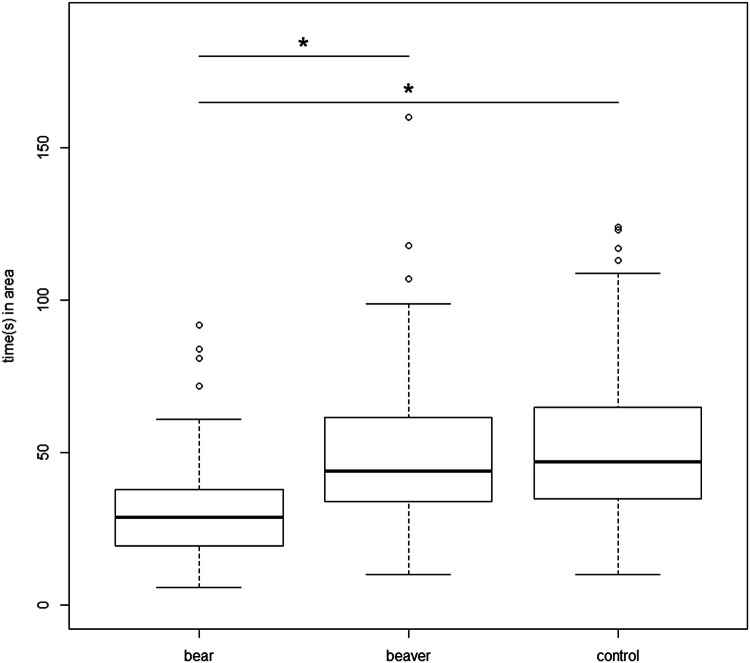
Amount of time (in seconds) spent in a 40-cm radius to each of the three bowls containing the bear, beaver, and control (water) scents by the dogs. “*” denotes a *p* value ≤ 0.05

On average, dogs spent 32.02 ± 19.31 (median = 29, range 6–92) seconds within the 40 cm radius of the bear faecal scent, 57.35 ± 52.97 (44, 10–380) seconds at the beaver faecal scent, and 54.33 ± 28.96 (47, 10–124) seconds at the control (Fig. 2). Time was log10 transformed to obtain normality for further analysis. We found that dogs spent significantly less time within the 40 cm radius of the bear faecal scent compared to the beaver (*β* = − 0.234, SE = 0.027, *df* = 108, *t* = 8.548, *p* ≤ 0.001) and control (*β* = − 0.244, SE = 0.027, *df* = 108, *t* = 8.905, *p* ≤ 0.001) scents. In addition, older dogs spent more time within the 40 cm radius of the scents than younger dogs (*β* = 0.022, SE = 0.008, *df* = 53, *t* = 2.771, *p* = 0.008). The variables sex, weight, hunting experience, and previous experience with bears were not significant and therefore removed from the analysis.

**Fig. 3:**
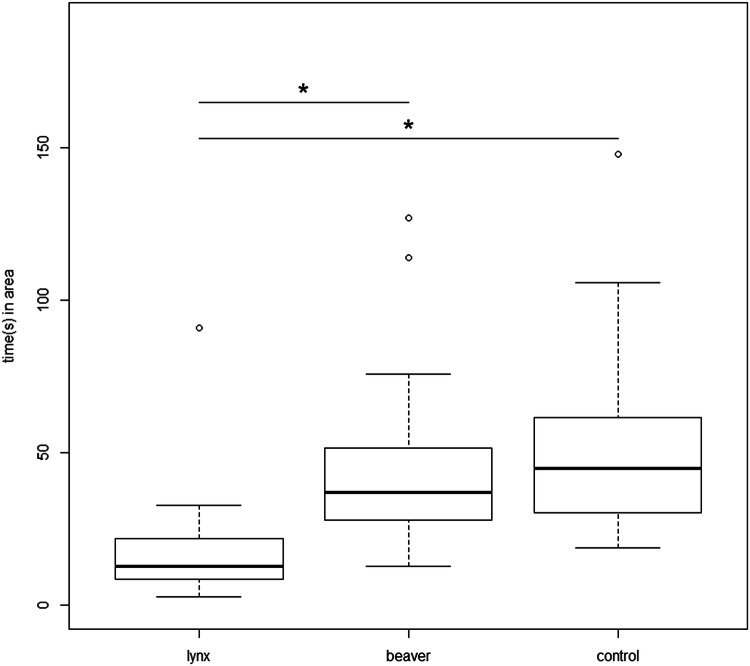
Amount of time (in seconds) spent in a 40-cm radius to each of the three bowls containing the lynx, beaver and control (water) scents by the dogs. “*” denotes a *p* value ≤ 0.05

On average, dogs spent 17.63 ± 16.85 (median = 13, range 3–91) seconds within the 40 cm radius of the lynx faecal scent, 43.44 ± 26.81 (37, 13–127) seconds at the beaver faecal scent, and 51.04 ± 29.81 (45, 19–148) seconds at the control (Fig. 3). Time was log10 transformed to obtain normality for further analysis. We found that dogs spent significantly less time within the 40 cm radius of the lynx faecal scent compared to the beaver (*β* = 0.446, SE = 0.051, *df* = 52, *t* = 8.706, *p* ≤ 0.001) and control (*β* = 0.518, SE = 0.051, *df* = 52, *t* = 10.113, *p* ≤ 0.001) scents. The variables sex, age, weight and use as hunting dog were not significant and therefore removed from the analysis.

### Change in heart rate

**Fig. 4:**
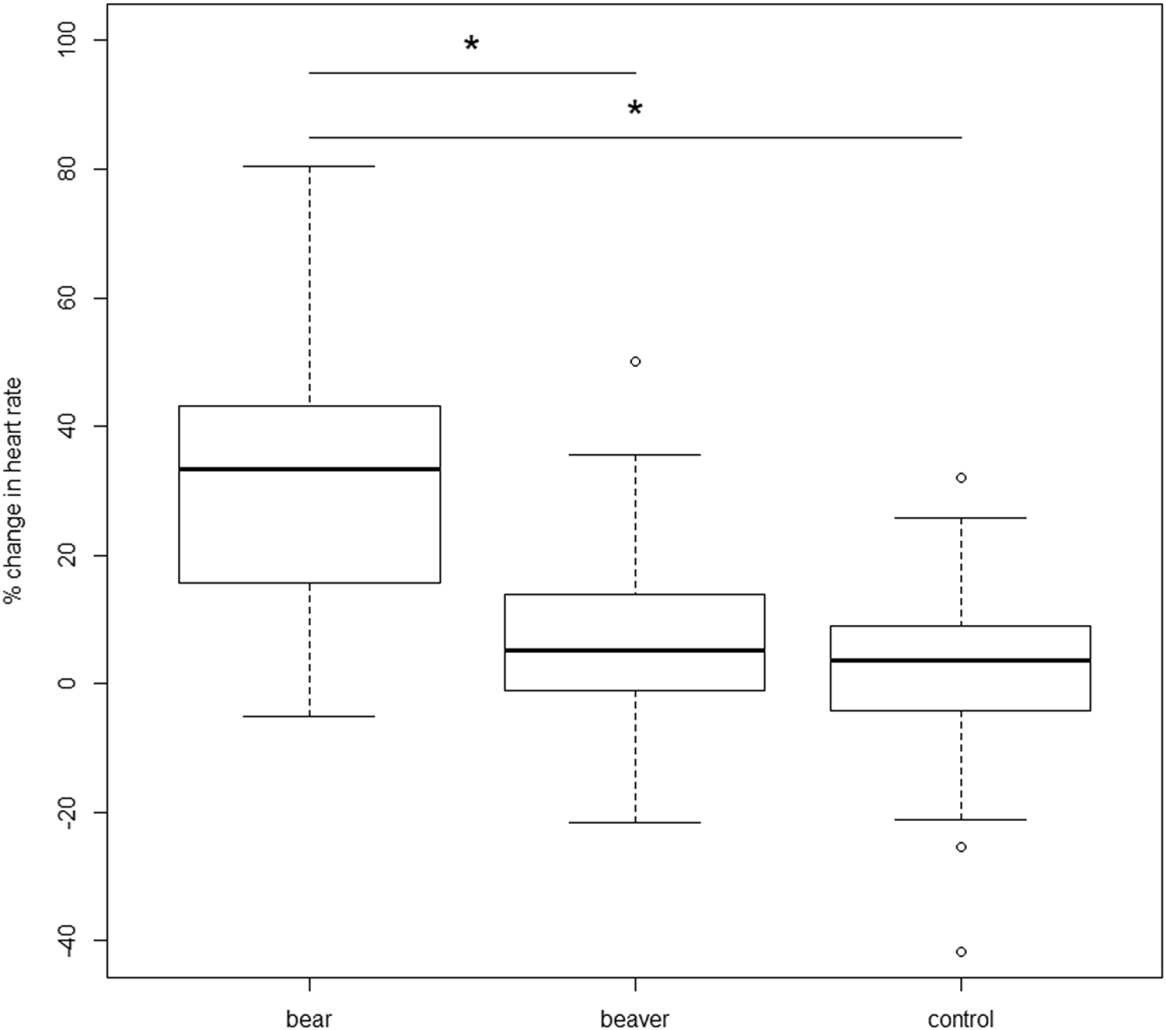
Proportional change in mean heart rate of each dog when in a 40-cm radius to each of the three bowls containing the bear, beaver and control (water) scents by the dogs. “*” denotes a *p* value ≤ 0.05

Dogs showed a mean proportional increase in heart rate of 30.03 ± 18.65% (33.3, − 5.1 to 81.0) within the 40 cm radius of the bear faecal scent, 6.72 ± 1.30% (5.1, − 21.6 to 50.0) at the beaver faecal scent, and 2.5 ± 13.2% (3.7, − 41.6 to 31.9) at the control (Fig. 4). We found that dogs had a significantly lower proportional increase in heart rate when within the 40 cm radius of the beaver faecal scent (*β* = − 0.233, SE = 0.020, *df* = 108, *t* = − 11.502, *p* ≤ 0.001) and the control scent (*β* = − 0.275, SE = 0.020, *df* = 108, *t* = − 13.588, *p* ≤ 0.001) compared to bear faecal scent. Female dogs showed a tendency to have a higher heart rate than male dogs (*β* = 0.060, SE = 0.033, *df* = 53, *t* = 1.830, *p* ≤ 0.073). The variables sex, age, weight, hunting experience, and previous experience with bear scent were not significant and therefore removed from the analysis.

**Fig. 5:**
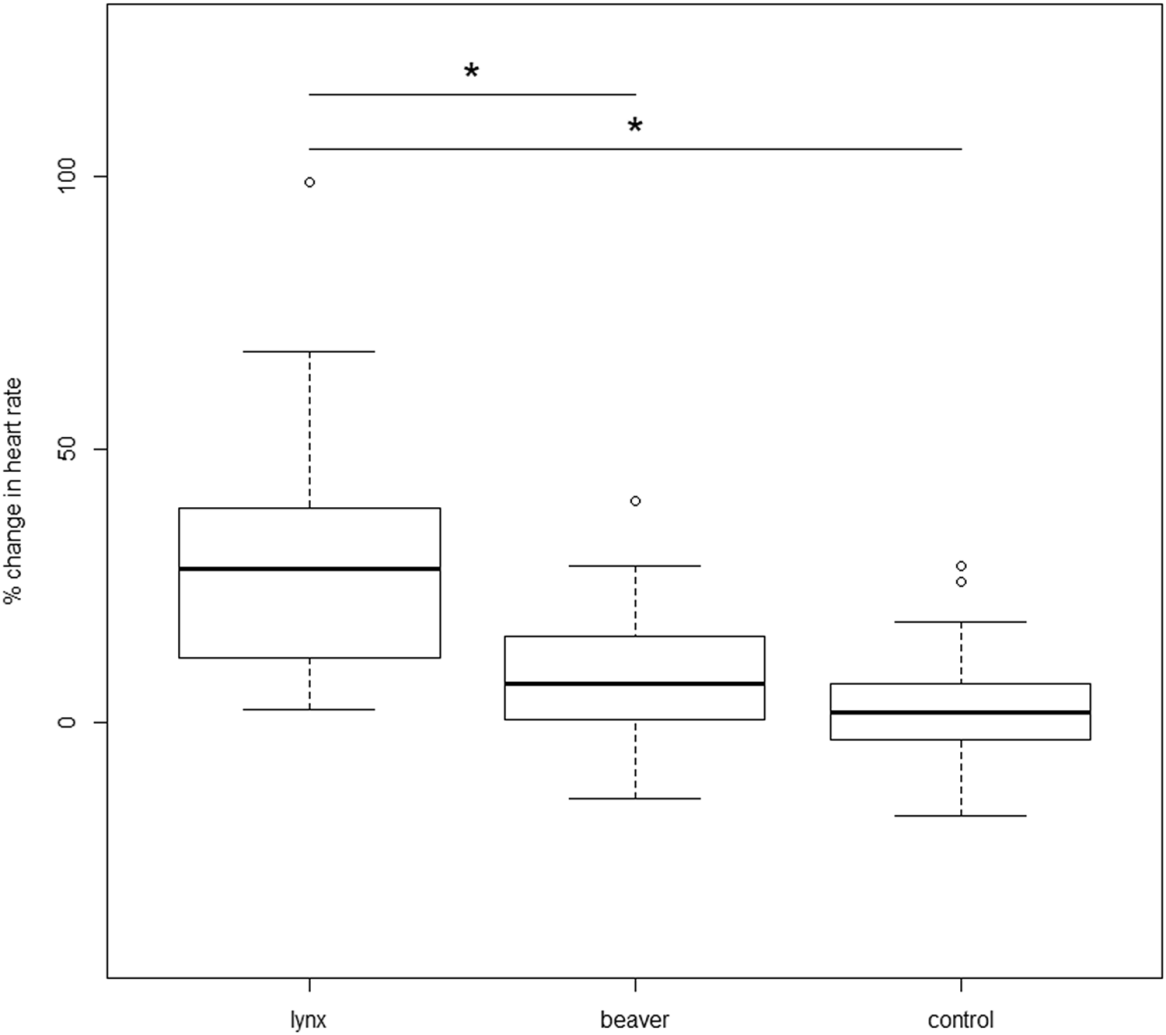
Proportional change in mean heart rate of each dog when in a 40-cm radius to each of the three bowls containing the lynx, beaver and control (water) scents by the dogs. “*” denotes a *p* value ≤ 0.05

Dogs showed a mean proportional increase in heart rate of 29.44 ± 22.25% (28.2, 2.4–99.1) within the 40 cm radius of the lynx faecal scent, 8.44 ± 12.58% (7.1, − 13.9 to 40.6) at the beaver faecal scent and 3.29 ± 11.11% (1.9, − 17.1 to 28.9) at the control (Fig. 5). We found that dogs had a significantly lower proportional increase in heart rate when within the 40 cm radius of the beaver faecal scent (*β* = 0.210, SE = 0.032, *df* = 52, *t* = − 6.470, *p* ≤ 0.001) and the control scent (*β* = − 0.261, SE = 0.032, *df* = 52, *t* = − 8.057, *p* ≤ 0.001) compared to the lynx faecal scent. The variables sex, age, weight and hunting experience were not significant and therefore removed from the analysis.

## Discussion

We showed that the exposure of domestic dogs to predator faecal odours of brown bear and Eurasian lynx affects the behavioural and physiological responses of the dogs, and therefore supported our hypotheses. Significantly less time was spent in the presence of the bear and lynx scents and a significantly higher mean heart rate relative to the dog’s basal heart rate was observed in response to the bear and lynx scents.

Sulphurous molecules found within the faeces of predators enable predator identification by prey species (Nolte et al. 1994). Arrays of sulphur-rich and nitrogen-rich volatile compounds may convey information to the receiver such as the type of food the predator has consumed (Cox et al. 2010), allowing the prey to alter their behaviour as a direct response. These behaviours are attributed to fear as seen within the study through the dogs spending a decreased length of time at the predator scents and an increase in heart rate around the scents. However, very little meat would have been present in the bear scats due to them being collected during fall (Stenset et al. 2016), so although the dogs could be reacting to the sulphurous metabolites present within the lynx scat this is not necessarily the case for the bear scats. This is, therefore, suggesting that something else is causing the increased heart rate and decreased time spent at the bear scent and suggestive of an innate reaction. Similar reactions have been seen in a study by Rosen et al. (2015) where an odour known as 2,5-dihydro-2,4,5-trimethylthiazoline (TMT) a known component of fox odours from anal gland secretions is seen to elicit threat-like properties in naïve mice (*Mus*). A further finding showed that dogs within the study spent an increased amount of time around the beaver scent compared to the predator scents. This again implies the dogs are having an innate reaction towards the predator scent, if they were altering their behaviour towards novel scents alone then they should also show a decreased amount of time spent around the beaver scent and an increased heart rate, but this was not seen within the study. A further finding showed the dogs spent a significantly decreased amount of time at the beaver bowl for the Norwegian data, although this was not true of the UK data. A likely reason for this behaviour is the dogs within the UK have had no previous experience with beavers nor their odours thus this is a novel scent, whereas within Telemark beavers are abundant in the river systems. Therefore, it is likely that the Norwegian dogs spent a decreased amount of time sniffing due to living sympatric with beavers (Rosell and Czech 2000), implying the scent is likely to be of less interest. The dogs that did show interest and chose to investigate the predator scent for shorter time periods could be related to the phenomenon known as ‘predator inspection’; which has been reported amongst a variety of taxa in both field and laboratory based studies (Fishman 1999). Some prey once they have detected an odour are seen to approach the scent, suggestive of the fact that the animal needs to examine the scent more closely to determine their response and the threat level. In our study, the dogs chose to spend a reduced amount of time at the lynx and bear scents. The dogs spent a reduced amount of time close to the scent and alongside total avoidance this is an effective strategy against potential predation within a wild setting. It decreases the probability of individuals being detected by the predator in question, through temporarily reducing exposure time (Martel and Dill 1993), which would limit potential predation and competition within the wild. The dogs within the study, however, are not in a wild setting and are domesticated therefore the reaction seen within the study are an innate reaction thus genetically manifested. The ‘Predation Risk Allocation’ hypothesis states that individuals should trade-off their foraging efforts in relation to temporal variation of predation risk, with foraging being lowest at the times of greatest risk (Lima and Bednekoff 1999), which has been seen within the study. The detection of a predator prior to an encounter via chemosensory cues is the first line of defence in the adaptive anti-predatory strategy and these cues are seen often to elicit avoidance (Haupt et al. 2010). Since a higher proportion of dogs avoid the lynx and bear scent or spend less time at those scents, there seems to be sensitive towards these scents. Therefore, dogs most likely have an innate response to the lynx and bear scents. These innate responses are likely to have resulted from a coexistence over evolutionary time, between the species (Ward et al. 1997) and the responses have been well documented in studies by for example Apfelbach et al. (2005) and Blumstein et al. (2002). However this is not just a simple predator–prey relationship, these three families of *Ursidae*, *Felidae* and *Canidae* are in a wild setting commonly seen to either compete for food or where possible avoid one another entirely. However although rare it is documented that members of the *Ursidae* and *Felidae* families are able to kill a single adult wolf and have been known to kill domestic dogs (Kojola and Kuittien 2002). During the domestication process prey animals may lose their predators and the associated pressures as a result of a relaxation in natural selection (Price 1999). Consequently, anti-predator behaviours may be reduced or lost (Blumstein 2006; Blumstein et al. 2006) as behaviours necessary for survival in a wild setting lose their adaptive significance (Price 1999). Predator-naïve prey can have a reduced sensitivity to stimuli that reveals the presence of predators (Berger et al. 2001). However, despite the domestication process for dogs as a species, this reaction to predators remains innate, as the bear scats were collected during fall where very little meat would be present but the dogs still reacted to these scents just as significantly as to the lynx scent showing that this is an innate reaction.

Predator species can have direct effects upon prey through the killing of individuals, but also indirect effects caused by fear (Altendorf et al*.* 2001), resulting in physiological stress (Matassa and Trussel 2014). Many animals are seen to use chemical cues from predators to first assess any risk of predation (Kats and Dill 1998). In addition to the stressful situation of a direct encounter of a potential predator, just the odours of a predator may act as a strong stressor (Hegab et al. 2014). From the overall results seen within this study, these physiological stresses within the dogs are true, as in all instances the presence of a lynx and bear scent suppressed the amount of time spent around the scent and an increase in heart rate of the dogs.

Physiological systems are activated during stressful and fear-inducing situations and heart rate is a good indicator to assess the emotional stress within dogs in these situations (Palestrini et al. 2005; Beerda et al. 1998) due to interactions in the central nervous system and neuroendocrine system. When the sympathetic nervous system is activated it increases blood pressure and heart rate (Hydbring-Sandberg et al. 2004). It has been suggested that a dogs heart rate increases non-specifically to stimuli and therefore the increase should be considered a general response to an event or change, irrespective of whether this is a positive or negative situation (Beerda et al. 1998). However, within the study, the basal heart rate was calculated during a 5 min interval when the dog was outside of the experimental room where the trials were conducted and exposed to just its owner and the experimenter, which in relation to the previous study would suggest an increased heart rate from just contact with its owner and a new individual (Palestrini et al. 2005). The dog’s heart rate however increased significantly when close to the predator scents. If a dog’s heart rate increased non-specifically it should increase at each of the scent bowls instead of just a significant response to the predator scent.

Many studies have evaluated the response of different species towards predator scents suggestive of responses being a result of experience, close phylogenetic relationships and even the compounds of the scents. However, in this study, none of these suggestions hold true and through dogs spending a decreased length of time and having an increased heart rate towards predator scents of brown bear and Eurasian lynx it suggests an innate reaction.

## Electronic supplementary material

Below is the link to the electronic supplementary material.

Supplementary file1 (DOCX 16 kb)
