# Supplementary material for: Fears from the past? The innate ability of dogs to detect predator scents
Source: Anim Cogn. 2020 Apr 8;23(4):721–9. doi: 10.1007/s10071-020-01379-y (PMC7320930; doi:10.1007/s10071-020-01379-y)
Supplement: Supplementary file 1 — Supplementary file1 (DOCX 16 kb) [file 10071_2020_1379_MOESM1_ESM.docx]

### A table to show information regarding each dog used within the trials.

| **Breed** | **Sex** | **Weight** | **Age** | **Hunting** | **Location** |
| --- | --- | --- | --- | --- | --- |
| Corgi | Male | 22 | 4 | No | Bø |
| Corgi | Female | 14 | 3 | No | Bø |
| Corgi | Female | 14 | 2 | No | Bø |
| Moose Hound | Male | 8 | 0.25 | Yes | Bø |
| Moose Hound | Male | 20 | 5 | Yes | Bø |
| English Springer Spaniel | Female | 24 | 11 | Yes | Bø |
| English Springer Spaniel | Female | 23 | 8 | Yes | Bø |
| English Springer Spaniel | Female | 22 | 3 | Yes | Bø |
| Golden Retriever mix | Male | 35 | 4 | No | Bø |
| German Short Haired Pointer | Male | 30 | 4 | Yes | Bø |
| Moose Hound | Male | 20 | 4 | Yes | Bø |
| Moose Hound | Male | 20 | 6 | Yes | Bø |
| Malamute | Male | 30 | 7 | No | Bø |
| Malamute | Male | 30 | 4 | No | Bø |
| Golden Retriever | Male | 22 | 0.5 | Yes | Bø |
| Australian Copper | Female | 18 | 2 | No | Bø |
| Border Collie mix | Male | 32 | 2 | No | Bø |
| Miniature schnauzer | Male | 8 | 9 | No | Bø |
| Border Collie | Male | 19 | 1 | No | Bø |
| Mixed Border Collie/Stover | Female | 28 | 6 | Yes | Bø |
| Moose Hound | Female | 14 | 2 | Yes | Bø |
| Basset Fauve de Bretagne | Female | 16 | 4 | Yes | Bø |
| Moose Hound | Female | 19 | 4 | Yes | Bø |
| Moose Hound | Female | 15 | 2 | Yes | Bø |
| Standard Poodle | Male | 24 | 8 | No | Bø |
| Standard Poodle | Female | 25 | 8 | No | Bø |
| Parson Russel Terrier | Female | 8 | 13 | No | Bø |
| Samoyed | Male | 32 | 8 | No | Bø |
| Papillion | Male | 3.4 | 2 | No | Bø |
| Papillion | Female | 3.6 | 2 | No | Bø |
| Shetland Sheepdog | Female | 8.5 | 2 | No | Bø |
| Samoyed | Female | 22 | 14 | No | Bø |
| Grosspitz | Male | 16 | 2 | No | Bø |
| Nova Scotia Duck Tolling Retriever | Male | 23 | 6 | No | Bø |
| English Springer Spaniel | Female | 18 | 0.75 | Yes | Bø |
| Petit basset Griffon Vendeen | Male | 15 | 5 | Yes | Bø |
| Border Collie | Female | 18 | 10 | No | Bø |
| Border Collie | Female | 22 | 12 | No | Bø |
| Border Collie | Male | 23 | 10 | No | Bø |
| Golden Retriever | Male | 34 | 7 | Yes | Bø |
| Samoyed | Male | 14 | 0.42 | No | Bø |
| Alaska Husky | Female | 22 | 9 | No | Bø |
| Alaska Husky | Female | 19 | 6 | No | Bø |
| German Shorthaired Pointer | Male | 28 | 4 | Yes | Bø |
| Kleiner Münsterlander | Male | 27 | 8 | Yes | Bø |
| Australian sheepdog | Male | 29 | 9 | No | Bø |
| Old English Sheepdog | Female | 23 | 9 | No | Bø |
| Australian Sheepdog | Male | 22 | 2 | No | Bø |
| Gordon setter | Male | 20 | 15 | No | Bø |
| Labrador mix | Female | 28 | 6 | No | Bø |
| Border Collie mix | Female | 18 | 7 | No | Bø |
| Golden Retriever | Male | 40 | 5 | No | Bø |
| Poodle | Male | 32 | 9 | No | Bø |
| Poodle | Female | 18 | 10 | No | Bø |
| Portuguese street dog | Male | 20 | 4 | No | Bø |
| Dachshund | Female | 7 | 2 | No | Ashby-de-la-Zouch |
| Yorkshire Terrier | Male | 6 | 7 | No | Ashby-de-la-Zouch |
| Patterdale Terrier | Male | 12 | 10 | No | Ashby-de-la-Zouch |
| Lurcher Cross Greyhound | Female | 17 | 11 | No | Ashby-de-la-Zouch |
| Lurcher Cross Bedlington Terrier | Female | 15 | 13 | No | Ashby-de-la-Zouch |
| Cairn Terrier | Male | 9 | 0.5 | No | Ashby-de-la-Zouch |
| Cocker Spaniel | Female | 15 | 8 | No | Ashby-de-la-Zouch |
| Springer Spaniel | Female | 7 | 0.33 | No | Ashby-de-la-Zouch |
| Springer Spaniel | Male | 24 | 9 | Yes | Ashby-de-la-Zouch |
| Springer Spaniel | Male | 17 | 3 | Yes | Ashby-de-la-Zouch |
| Cavalier King Charles Spaniel | Female | 8 | 1 | No | Ashby-de-la-Zouch |
| Bearded Collie | Male | 36 | 6 | No | Ashby-de-la-Zouch |
| Ladradoodle | Male | 22 | 6 | No | Ashby-de-la-Zouch |
| German Shepard mix | Male | 35 | 4 | No | Ashby-de-la-Zouch |
| Labrador | Female | 25 | 4 | No | Ashby-de-la-Zouch |
| Springer/Cocker Spaniel | Female | 14.4 | 3 | Yes | Ashby-de-la-Zouch |
| Labrador | Male | 28 | 4 | No | Ashby-de-la-Zouch |
| Border Collie | Male | 23 | 3 | no | Ashby-de-la-Zouch |
| Cavalier King Charles Spaniel | Male | 10 | 6 | No | Ashby-de-la-Zouch |
| Cocker/field Spaniel | Male | 13.5 | 1 | Yes | Ashby-de-la-Zouch |
| Cavalier King Charles Spaniel | Male | 9 | 6 | No | Ashby-de-la-Zouch |
| Springer/Cocker Spaniel | Female | 13.5 | 2 | Yes | Ashby-de-la-Zouch |
| Springer Spaniel | Female | 4.1 | 0.33 | No | Ashby-de-la-Zouch |
| West highland Terrier | Male | 9 | 9 | No | Ashby-de-la-Zouch |
| Beagle | Male | 18 | 6 | Yes | Ashby-de-la-Zouch |
| Labrador | Male | 30 | 7 | No | Ashby-de-la-Zouch |
| Border Terrier | Male | 10 | 9 | No | Ashby-de-la-Zouch |
